# Demographic and Socioeconomic Influences on Sleep Patterns among Adolescent Students

**DOI:** 10.3390/ijerph17124378

**Published:** 2020-06-18

**Authors:** Jinseok Kim, Jin-Won Noh, Ahraemi Kim, Young Dae Kwon

**Affiliations:** 1 Department of Social Welfare, Seoul Women’s University, Seoul 01797, Korea; jskim@swu.ac.kr (J.K.); akim@swu.ac.kr (A.K.); 2 Department of Health Administration, Dankook University, Cheonan 31116, Korea; health@dankook.ac.kr; 3 Global Health Unit, Department of Health Sciences, University Medical Centre Groningen, University of Groningen, 9713 GZ Groningen, The Netherlands; 4 Department of Humanities and Social Medicine, College of Medicine and Catholic Institute for Healthcare Management, The Catholic University of Korea, Seoul 06591, Korea

**Keywords:** demographic factors, socioeconomic factors, sleep patterns, adolescent students, Korean Children and Youth Panel Survey

## Abstract

Although proper sleep is an important topic in adolescent health, little is known about the sleep patterns of adolescents from a longitudinal and non-Western perspective. To fill this gap, the present research conducted a longitudinal study of the impact of demographic and socioeconomic factors on sleep patterns among Korean adolescent students. The relationship could positively or negatively affect sleep. Therefore, it is important to understand which demographic and socioeconomic factors are related to sleep patterns. This study used nationally representative panel data from the Korean Children and Youth Panel Survey. A series of descriptive analyses were conducted to provide overall characteristics of the sample. Furthermore, mixed effect regression analysis techniques were employed to test the relationship between demographic and socioeconomic factors and sleep patterns. Paternal employment status was associated with adolescent sleep patterns, while maternal employment status was not. Adolescents with both parents working compared to adolescents with one parent or none working showed different sleep patterns on weekdays but not on weekends. Both parents possessing college degrees, household income, living in an urban area, and family type were associated with adolescent sleep pattern indicators to varying degrees. Some of these associations varied according to adolescent sex. This study provides insight into the impact of demographic and socioeconomic factors on weekend and weekday sleep patterns among adolescent students by sex. These findings provide information for the promotion of healthy sleep in adolescents by addressing demographic and socioeconomic factors.

## 1. Introduction

Proper sleep of adolescents has been regarded as an essential health component. Sleep problems are linked to both physical and mental health outcomes including obesity [1,2] and substance abuse [3]. Thus, promotion of proper sleep time in adolescents is a crucial public health concern. However, sleep problems have been globally prevalent in adolescents [4]. Due to adolescent time schedules, sleep deficiency on weekdays and oversleep on weekends are likely [5]. In addition, internet use and high enthusiasm for education aggravate sleep problems [6,7]. Sleep research is especially important because adolescent sleep patterns are prone to being irregular and insufficient.

In light of this, adolescent sleep has received a great deal of attention from researchers. Researchers have studied sleep patterns (e.g., sleep duration, rise time, and bedtime) [6,8,9,10]. Some studies only focused on identifying optimal hours of sleep [11]. A large number of researchers have extended their interests to sleep patterns including total sleep time, rise time, and bedtime [12]. Also, some studies considered the difference in sleep patterns between weekdays and weekends. However, the research on sleep patterns is more likely to utilize cross-sectional designs due to limited access to longitudinal data; a longitudinal study of adolescent sleep patterns is necessary. Examining the longitudinal data would be beneficial for researchers and other professionals to better understand not only adolescents’ sleep patterns but also the overall trend of sleep behaviors of this fast-growing population.

The association between adolescent sleep patterns and demographic and socioeconomic factors has previously been established [13,14,15,16,17]. The sex of adolescents has been included in most of the relevant studies as sleep patterns are different according to sex [16,17]. Single-parent-family adolescents were more likely to have poor sleep patterns [15,18,19], and having sibling(s) may result in later bedtimes [13,16]. Educational level and working status of parents were negatively associated with adolescent sleep patterns [15,20]. Low household income was linked to poor sleep patterns [17,18,19]. Adolescent sleep patterns may differ depending on rural or urban social environments [19].

Diverse demographic and socioeconomic factors affect adolescent sleeping environments, and inclusion of these factors in adolescent sleep research is essential because adolescents’ sleep can affect their physical health and mental health in a variety of ways. Prior research studies had limitations in that the associated factors were examined from a cross-sectional perspective in limited cultural contexts [15,17]. In response to this research gap, this study aimed to examine the impact of demographic and socioeconomic factors on sleep patterns among adolescent students using nationally representative longitudinal data from Korea.

## 2. Materials and Methods

### 2.1. Data and Participants

This study used data from the Korean Children and Youth Panel Survey (KCYPS). The KCYPS is a nationally representative study of Korean children and youths with a focus on various facets of growth and development. The KCYPS is comprised of three different panels of students: first- and fourth-grade elementary students and first-year junior high school students in 2010 (*n* = 7071). These students were followed annually until 2016. The KCYPS utilized a multistage stratified cluster sampling method with schools as the primary sampling unit. This specific study analyzed the panel data from the junior high school students (*n* = 2351) from their first year of junior high school (wave 1) to the last year of high school (wave 6). Participating adolescents’ parents or other guardians such as grandparents also answered questions related to household characteristics such as family type, parents’ working status, and household income, etc. This study was approved by the Institutional Review Board of the Seoul Women’s University (IRB-2018-46) with a waiver for informed consent because the data were obtained from a public data depository which is freely accessible online at http://archive.nypi.re.kr/modedg/contentsView.do?ucont_id=CTX000029&menu_nix=qZc474Ak.

### 2.2. Variables and Measurements

The adolescent students’ sleep patterns were measured using three indicators of bedtime, wake-up time, and sleep duration on weekdays and weekend days. The sleep duration variable was calculated using the measure of bedtime and wake-up time variables. Bedtime and wake-up time were measured using the answers to the following question: “What time do you usually fall asleep and get up?” This question was asked for weekdays and for weekend days separately.

A series of variables measuring adolescent demographic and socioeconomic characteristics were used in this analysis. Adolescent demographic characteristics were measured by sex and family structure. The family structure variable was measured using two different variables: whether the student had siblings and the type of family. The type of family was defined as living with “both parents”, “one parent”, or “others” (e.g., living with grandparents). Adolescent socioeconomic statuses were measured by parent working status, parent education level, household income, and area of living. Parental work statuses were acquired from parents and used as both separate and combined variables; the combined variable demonstrated that both parents were working. Education levels of mothers and fathers were defined by attainment of college degrees. A question about household income was included in the parent questionnaire of the survey and used as a quantitative measure. Area of living was defined as whether an adolescent lived in an urban or rural area.

### 2.3. Statistical Analysis

A series of descriptive analyses were conducted to provide the overall characteristics of the sample. Considering the repeated measure of the panel data, mixed effect regression analysis techniques were utilized to test the relationship between sleep patterns and adolescent demographic and socioeconomic characteristics [21]. Furthermore, the analyses of the relationship between adolescent student sleep patterns and various demographic and socioeconomic variables were conducted for the total sample and in a sex-stratified manner. Stata 15 (StataCorp LP, College Station, TX, USA) was used to manage the data and to calculate the model.

## 3. Results

The overall characteristics of the sample across the entire study period are shown in Table 1. The study participants were evenly divided in terms of sex in wave 1. Approximately 20% of adolescent students were not from two-parent families, and approximately 90% of adolescents had one or more sibling(s). Approximately two-thirds of mothers and over 90% of fathers were working throughout the study periods, and 60% to 70% of adolescent students in this study were from households in which both parents were working. The proportion of mothers with college degrees ranged from 26% to 33% during the study period, whereas that of fathers ranged from 41% to 46%. Finally, about 85% of participants were living in urban areas. Table 1 summarizes the sleep-pattern-related variables of adolescent students. These students reported from 6.12 to 7.90 h of sleep on weekdays during the study period. Overall, older Korean adolescent students spent less time sleeping. When compared to their younger age, respondent bedtimes became later with age but rising time did not change as much as the bedtime did (Table 1).

The results of the analysis on the relationship between various measures of adolescent student sleep patterns including sleep duration, bedtime, and wake-up time on weekdays and weekends and their demographic and socioeconomic variables are summarized in Table 2. Family-structure-related variables were associated with sleeping patterns of adolescent students. Adolescents of one-parent families (B (SE) = 7.204 (2.736) *p* = 0.008) and other family type, i.e., adolescents living with family members other than parents (B (SE) = 9.285 (2.663) *p* < 0.001) slept longer than adolescents living with two parents on weekdays but not on weekends. The same relationship was found for males (one-parent family: B (SE) = 11.878 (3.755), *p* = 0.002; other: B (SE) = 9.676 (3.685), *p* = 0.009) but not for females. However, whether adolescents had any siblings was not associated with any sleep pattern indicators used in this study (Table 2).

Results showed that the mother’s working status was not associated with any sleep pattern measures for any sex-stratified group. However, adolescents of working fathers reported later bedtimes (B (SE) = 8.231 (3.021), *p* = 0.006) and earlier wake-up times (B (SE) = −10.073 (2.411), *p* < 0.001) during weekdays and slept less than students of nonworking fathers on both weekdays (B (SE) = −19.878 (3.432), *p* < 0.001) and weekends (B (SE) = −12.830 (4.490), *p* = 0.004). This sleep duration relationship was found for both female (weekdays: B (SE) = −21.354 (4.917), *p* < 0.001; weekends: B (SE) = −13.469 (6.484), *p* = 0.038) and male (weekdays: B (SE) = −18.090 (4.729), *p* < 0.001; weekends: B (SE) = −12.811 (6.171), *p* = 0.038) students. Furthermore, when both parents were working, adolescents tended to rise earlier (B (SE) = −3.541 (1.142), *p* = 0.002) and to sleep less (B (SE) = −4.246 (1.664), *p* = 0.011) on weekdays but not on weekends compared to their counterparts. The same relationship was found for males only (wake-up time: B (SE) = −3.575 (1.624), *p* = 0.028; sleep duration: B (SE) = −5.383 (2.337), *p* = 0.021).

Mother’s attainment of a college degree was associated with an adolescent having a later bedtime (B (SE) = 11.878 (1.633), *p* < 0.001) and shorter sleep duration (B (SE) = −13.976 (1.777), *p* < 0.001) on weekdays. The same was true for father’s attainment of a college degree (bedtime: B (SE) = 12.577 (1.555), *p* < 0.001; sleep duration: B (SE) = −12.583 (1.699), *p* < 0.001). On weekends, mothers with a college degree (B (SE) = −17.327 (2.463), *p* < 0.001) and fathers with a college degree (B (SE) = −13.297 (2.350), *p* < 0.001) were associated with shorter adolescent sleep durations due to earlier wake-up time (mothers with a college degree: B (SE) = −13.042 (2.633), *p* < 0.001; fathers with a college degree: B (SE) = −9.018 (2.508), *p* < 0.001). Similar patterns of relationship were found for both the male (mothers with a college degree on weekday sleep duration: B (SE)= −11.540 (2.511), *p* < 0.001; that on weekend sleep duration: B (SE) = −14.353 (3.473), *p* < 0.001) and female groups (mothers with a college degree on weekday sleep duration: B (SE) = −16.292 (2.459), *p* < 0.001; that on weekend sleep duration: B (SE) = −20.306 (3.449), *p* < 0.001).

Higher household income was also associated with shorter adolescent student sleep durations (B (SE) = −0.003 (<0.001), *p* < 0.001) and with later bedtimes (B (SE) = 0.003 (<0.001), *p* < 0.001) but not with wake-up time on weekdays. On weekends, there were associations with all three sleep indicators (sleep duration: B (SE) = −0.003 (<0.001), *p* < 0.001; bedtime: B (SE) = 0.001 (<0.001), *p* < 0.001; wake-up time: B (SE) = −0.001 (<0.001), *p* = 0.011). Similar patterns of relationship between household income and sleep indicators were found for both female only (weekday sleep duration: B (SE) = −0.003 (<0.001), *p* < 0.001; weekday bedtime: B (SE) = 0.003 (<0.001), *p* < 0.001; weekday wake-up time: B (SE) < 0.000 (<0.001), *p* = 0.541; weekend sleep duration: B (SE) = −0.003 (<0.001), *p* < 0.001; weekend bedtime: B (SE) = 0.001 (<0.001), *p* = 0.003; weekend wake-up time: B (SE) = −0.002 (0.001), *p* = 0.004) and male only samples (weekday sleep duration: B (SE) = −0.003 (<0.001), *p* < 0.001; weekday bedtime: B (SE) = 0.003 (<0.001), *p* < 0.001; weekday wake-up time: B (SE) < 0.000 (<0.001), *p* = 0.367; weekend sleep duration: B (SE) = −0.002 (0.001), *p* < 0.001; weekend bedtime: B (SE) = 0.002 (<0.001), *p* = 0.001; weekend wake-up time: B (SE) = −0.001 (0.001), *p* = 0.379).

Adolescent students who were living in urban areas had later bedtimes and woke up later than students in rural areas on weekdays (bedtime: B (SE) = 12.184 (2.191), *p* < 0.001; wake-up time: B (SE) = 3.596 (1.497), *p* = 0.016) and weekends (bedtime: B (SE) = 12.094 (2.674), *p* < 0.001; wake-up time: B (SE) = 9.811 (3.529), *p* = 0.005). However, sleep duration was shorter when adolescents were living in urban areas compared to rural areas on weekdays (B (SE) = −9.041 (2.374), *p* < 0.001), but the same was not true on weekends. Similar patterns were found for both males (weekday sleep duration: B (SE) = −10.261 (3.479), *p* < 0.001; weekday bedtime: B (SE) = 15.723 (3.227), *p* < 0.001; weekday wake-up time: B (SE) = 5.328 (2.216), *p* = 0.016; weekend sleep duration: B (SE) = −5.973 (4.787), *p* = 0.212; weekend bedtime: B (SE) = 12.286 (4.109), *p* = 0.003; weekend wake-up time: B (SE) = 6.024 (5.197), *p* = 0.246) and females (weekday sleep duration: B (SE) = −9.233 (3.156), *p* = 0.003; weekday bedtime: B (SE) = 9.807 (2.968), *p* = 0.001; weekday wake-up time: B (SE) = 1.335 (1.982), *p* = 0.501; weekend sleep duration: B (SE) = 2.724 (4.471), *p* = 0.542; weekend bedtime: B (SE) = 12.060 (3.466), *p* = 0.001; weekend wake-up time: B (SE) = −14.145 (4.706), *p* = 0.003) (Table 2).

## 4. Discussion

Adolescent sleep patterns are affected by a variety of parental and household economic, social, cultural, and environmental indicators [22,23]. Adolescent sleep pattern and duration vary across countries [4]. The Republic of Korea has experienced rapid socioeconomic growth over the past several decades, and this has led to a substantial transformation of lifestyles in the country [24]. Asian adolescents, including Korean students, are more likely to have later bedtimes than adolescents from Western countries, resulting in less total sleep time [4].

This study examined the impact of the demographic and socioeconomic factors on sleep patterns among adolescent students using longitudinal data representative of the Korean population. Using longitudinal data is important for researchers and other professionals to better understand not only adolescents’ sleep pattern trend but also the overall time trend of sleep behaviors of this fast-growing and changing population. Family structures were associated with sleep patterns in this study. Adolescent students living with one parent or other family members experienced longer sleep duration on weekdays. The same relationship was found for male adolescents. The sex difference in the association between being in a one-parent household and sleep duration on weekdays may be the result of sex differences in self-control. That is, females are known to show higher levels of self-control than male [25]; females, therefore, are less likely to be affected by family structure than males. Prior studies have reported inconsistent results for this relationship. One study reported no association between parental marital status and weekend or weekday sleep duration in adolescent students [26]. Another study reported that adolescents living with single parents had shorter sleep duration on weekends than adolescents with two parents [27]. This study result can be explained by Korean adolescent students having considerable private education. Single-parent households, however, may be less focused on their student’s private education because single parents have been shown to place less pressure on their children’s educational achievement than two parents [25]. This would allow adolescent students living with one parent or other family members to have more time for sleep on weekdays.

Engagement of both parents in work is associated with some poor sleep behaviors in adolescents. However, sex differences were seen in this relationship. Having a working father was associated with shorter sleep duration in this study. Although sex roles have changed considerably in recent decades, the majority of mothers worked part-time whereas most fathers worked full-time or longer [28]. Moreover, Korean male employees have the longest workday in the world [29]. Having a father present in the household is associated with a decreased likelihood of having a regular bedtime. The presence of a father may be associated with parents wanting to have the child awake when either or both parents return home for the day [15]. Similarly, these students may rise earlier to be present when their father leaves for work. Also, adolescents could be affected by the early-rising parent’s alarm clock. Therefore, adolescents with working fathers tend to rise earlier and to consequently sleep for shorter periods. When both parents were working, adolescent students tended to rise earlier and to sleep less on weekdays. The same relationship was found for males only. The sex difference in adolescents is also demonstrated in this study. The difference may be due to male adolescents being more affected by the family environment than female adolescents [30].

Attainment of college degrees by either or both parents was associated with short adolescent student sleep durations. Korean emphasis on educational achievement is especially strong [31]. Adolescents with better-educated parents may spend more time studying [32]. On weekdays, higher educated parents might expect their children to study later even if their children need to attend school the next day. On weekends, maintenance of weekday routines may be a reason for early adolescent arousal.

Higher household income was associated with shorter adolescent student sleep duration and with later bedtime. A higher prevalence of sleep problems in adolescents from higher socioeconomic status households is a consistent pattern previously observed in the US and elsewhere [33,34]. Parents with higher socioeconomic statuses reported more concern about inadequate sleep in their children [35]. Higher socioeconomic status may be associated with adolescent use of the internet, television watching, playing video games, and having heavier homework loads, all of which shorten sleep duration.

Adolescent students living in urban areas had later bedtimes and wake-up times than those living in rural areas. Adolescent students living in urban areas had shorter sleep duration than those living in rural areas. These differences in this research may be the result of social environment differences between urban and rural areas [19]. Urban adolescents may have more night activities than rural adolescents. This includes more opportunities to access electronic media and/or the internet because of social and economic developmental disparities between rural and urban areas [36]. Increased electronic media use may shorten sleep duration from later bedtimes and may decrease sleep quality by contributing to increased arousal and disruptions in the circadian regulation of sleep caused by artificial light [37].

There are potential limitations in this study. First, self-reported data on sleep patterns may not provide accurate information due to recall bias. Second, potential confounding effects on sleep patterns such as those associated with sleep quality and current health status of adolescent students were not adequately considered due to the lack of data. Last, this sample consisted of school students; the results may not be completely generalizable to the entire Korean adolescent population. However, to the best of our knowledge, this study is the first study to find associations between sleep patterns on both weekdays and weekends and demographic and socioeconomic factors by sex using national representative data.

## 5. Conclusions

Sleep patterns are associated with multiple health risks in adolescents. This study provides insight into the impact of the demographic and socioeconomic factors on sleep patterns on weekends and weekdays among adolescent students by sex. These findings provide important information for the promotion of healthy sleep in adolescent students by addressing demographic and socioeconomic factors. Further research is necessary to identify the ecological pathways that lead to poor sleep patterns in adolescents and to investigate the consequences of poor sleep patterns on adolescent physical and psychological health.

## Figures and Tables

**Table 1:** Characteristics of the sample of adolescent students (*n* = 2351).

Variable	Category	Wave 1		Wave 2		Wave 3		Wave 4		Wave 5		Wave 6	
		*n*	%	*n*	%	*n*	%	*n*	%	*n*	%	*n*	%
Sex	Female	1176	50.0										
Family structure	Total												
	Single parent	224	9.5	174	7.9	203	9.3	192	9.3	189	9.4	178	9.2
	Others	288	12.3	229	10.4	187	8.5	160	7.8	148	7.4	137	7.1
	Female												
	Single parent	111	9.5	83	7.7	98	9.0	91	9.0	85	8.7	87	9.1
	Others	140	11.9	111	10.3	93	8.6	75	7.4	70	7.2	59	6.2
	Male												
	Single parent	113	9.6	91	8.1	105	9.5	101	9.7	104	10.1	91	9.2
	Others	148	12.6	118	10.6	94	8.5	85	8.1	78	7.6	78	7.9
Have any sibling	Total	2149	91.4	2001	87.8	1999	88.5	1885	89.4	1837	87.9	1765	85.9
	Female	1082	92.1	996	88.3	993	88.7	935	90.5	899	87.8	875	86.2
	Male	1067	90.7	1005	87.2	1006	88.3	950	88.4	938	87.9	890	85.5
Mother working	Total	1376	62.5	1386	67.3	1407	68.5	1321	68.7	1332	70.8	1257	65.2
	Female	711	63.8	696	67.6	717	69.6	666	69.3	674	72.2	635	66.8
	Male	665	61.1	690	66.9	690	67.3	655	68.0	658	69.5	622	63.7
Father working	Total	2101	96.5	2002	98.5	1975	97.3	1861	97.4	1802	97.5	1741	90.4
	Female	1050	96.5	993	98.7	985	98.0	922	98.0	879	97.6	856	90.2
	Male	1051	96.5	1009	98.3	990	96.7	939	96.9	923	97.4	885	90.6
Both parents working	Total	1211	59.1	1256	64.9	1250	65.2	1193	66.1	1190	68.0	1130	59.0
	Female	623	60.3	634	65.6	642	67.0	607	67.5	604	69.6	570	60.3
	Male	588	57.9	622	64.3	608	63.3	586	64.8	586	66.4	560	57.7
Mother with college degree	Total	571	26.0	670	32.7	666	32.3	617	32.1	614	32.7	586	32.3
	Female	290	26.2	338	32.9	335	32.5	319	33.2	308	33.1	303	33.3
	Male	281	25.9	332	32.4	331	32.2	298	30.9	306	32.3	283	31.3
Father with college degree	Total	883	40.7	934	46.2	915	45.1	849	44.5	834	45.1	787	44.3
	Female	449	41.5	464	46.2	454	45.2	420	44.7	403	45.0	389	44.6
	Male	434	40.0	470	46.1	461	45.0	429	44.3	431	45.3	398	44.0
Living area: urban	Total	2014	85.7	1876	85.4	1929	85.4	1801	85.4	1721	85.7	1629	84.1
	Female	989	84.2	908	84.2	942	84.2	872	84.4	819	84.0	786	82.5
	Male	1025	87.2	968	86.5	987	86.6	929	86.4	902	87.3	843	85.6
		Mean	SD	Mean	SD	Mean	SD	Mean	SD	Mean	SD	Mean	SD
Household income (million Korean won/year)	Total	43.6	28.6	45.2	24.8	46.8	27.2	46.7	26.4	46.9	25.5	48.1	26.0
	Feale	43.1	26.6	45.7	24.9	47.4	26.6	47.3	25.7	47.2	25.7	48.6	26.0
	Male	44.2	30.4	44.7	24.7	46.2	27.8	46.1	27.0	46.6	25.3	47.6	25.3
Sleep duration during weekdays (h)	Total	7.90	0.98	7.67	0.96	7.36	1.01	6.31	1.10	6.12	1.12	6.31	1.21
	Female	7.75	0.96	7.48	0.95	7.19	1.02	6.16	1.06	6.01	1.08	6.20	1.16
	Male	8.05	0.98	7.86	0.94	7.52	0.97	6.46	1.11	6.22	1.15	6.43	1.24
Bedtime during weekdays (h)	Total	23.12	0.93	23.34	0.93	23.66	0.99	24.28	1.04	24.57	1.05	24.51	1.12
	Female	23.23	0.94	23.47	0.94	23.77	1.00	24.34	1.03	24.60	1.02	24.53	1.08
	Male	23.01	0.91	23.22	0.91	23.56	0.96	24.22	1.04	24.55	1.08	24.50	1.15
Rise-time during weekdays (h)	Total	7.02	0.48	7.01	0.48	7.02	0.51	6.59	0.51	6.69	0.56	6.83	0.77
	Female	6.97	0.44	6.95	0.47	6.96	0.48	6.51	0.50	6.61	0.54	6.73	0.71
	Male	7.07	0.51	7.07	0.48	7.08	0.53	6.68	0.50	6.76	0.57	6.93	0.81
Sleep duration during weekends (h)	Total	9.52	1.57	9.40	1.59	9.14	1.61	8.53	1.64	8.32	1.63	8.25	1.73
	Female	9.74	1.63	9.61	1.62	9.30	1.55	8.69	1.65	8.40	1.64	8.26	1.70
	Male	9.30	1.48	9.20	1.53	8.98	1.65	8.37	1.61	8.25	1.61	8.24	1.76
Bedtime during weekends (h)	Total	23.76	1.17	23.90	1.19	24.35	1.29	24.86	1.28	25.10	1.25	25.17	1.36
	Female	23.90	1.18	24.05	1.17	24.45	1.28	24.84	1.23	25.07	1.17	25.12	1.27
	Male	23.63	1.15	23.75	1.19	24.25	1.29	24.88	1.34	25.12	1.32	25.22	1.44
Rise-time during weekends (h)	Total	9.28	1.64	9.30	1.64	9.49	1.65	9.39	1.65	9.42	1.69	9.42	1.82
	Female	9.64	1.65	9.65	1.65	9.75	1.57	9.53	1.63	9.47	1.64	9.38	1.74
	Male	8.92	1.55	8.96	1.55	9.23	1.69	9.26	1.66	9.37	1.74	9.46	1.89

Note:SD, standard deviation. The participants were in their 1st year of junior high school at wave 1 and in the last year of high school at wave 6.

**Table 2:** Relationship between socioeconomic and demographic characteristics and sleep patterns of adolescent students.

	Weekdays						Weekends					
	Sleep Duration		Bedtime		Get-Up Time		Sleep Duration		Bedtime		Get-Up Time	
	B	SE (B)	B	SE (B)	B	SE (B)	B	SE (B)	B	SE (B)	B	SE (B)
Family structure: one parent	7.204 **	2.736	−4.243 *	2.473	1.800	1.802	4.557	3.710	6.326 *	3.006	9.733 *	3.902
Family structure: other	9.285 ***	2.663	−6.130 **	2.363	2.537	1.807	2.270	3.534	−0.614	2.853	1.205	3.661
Female	1.803	3.897	−2.000	3.608	−0.026	2.525	8.035	5.391	7.267 ^+^	4.153	14.493 **	5.580
	8.789 *	3.780	−5.595	3.430	2.781	2.534	11.552 *	5.072	−4.254	3.877	7.222	5.150
Male	11.878 **	3.755	−5.893 *	3.370	3.184	2.525	1.471	5.060	5.373	4.321	5.370	5.365
	9.676 **	3.685	−6.402 *	3.241	2.066	2.538	-6.103	4.882	2.825	4.156	−4.086	5.115
Have any sibling	−0.456	2.382	2.163	2.139	1.677	1.576	1.809	3.205	2.191	2.607	4.327	3.351
Female	−0.706	3.386	0.915	3.106	−1.158	2.206	1.450	4.605	−0.862	3.535	1.259	4.726
Male	0.363	3.283	2.851	2.932	4.676 *	2.206	1.754	4.421	5.404	3.799	7.083	4.665
Mother working	−1.738	1.662	−0.255	1.480	−1.931	1.124	−1.553	2.211	2.053	1.782	−0.113	2.307
Female	1.545	2.331	−1.892	2.134	−0.543	1.551	−2.489	3.162	2.307	2.411	−1.115	3.236
Male	−4.198	2.329	0.997	2.044	−2.857	1.597	−1.321	3.066	1.711	2.612	0.016	3.234
Father working	−19.878 ***	3.432	8.231 **	3.021	−10.073 ***	2.411	−12.830 **	4.490	2.360	3.622	−10.514 *	4.630
Female	−21.354 ***	4.917	6.991	4.432	−12.834 ***	3.407	−13.469 *	6.484	0.738	4.956	−12.427 *	6.543
Male	−18.090 ***	4.729	8.823 *	4.101	−7.172 *	3.373	−12.811 *	6.171	3.706	5.245	−9.235	6.443
Both parents working	−4.246 *	1.664	0.524	1.477	−3.541 **	1.142	−1.878	2.195	0.385	1.769	−2.022	2.284
Female	−2.242	2.330	−0.855	2.123	−3.027	1.575	−2.024	3.122	0.763	2.384	−2.046	3.184
Male	−5.383 *	2.337	1.458	2.047	−3.575 *	1.624	−2.469	3.058	−0.143	2.601	−2.914	3.217
Father with college degree	−12.583 ***	1.699	12.577 ***	1.555	1.023	1.087	−13.297 ***	2.350	4.111 *	1.905	−9.018 ***	2.508
Female	−14.226 ***	2.364	15.766 ***	2.204	2.556 *	1.493	−15.541 ***	3.336	7.209 **	2.587	−8.523 *	3.491
Male	−10.913 ***	2.378	9.248 ***	2.178	−0.407	1.543	−11.616 ***	3.267	0.621	2.781	−10.570 **	3.527
Mother with college degree	−13.976 ***	1.777	11.878 ***	1.633	−1.328	1.161	−17.327 ***	2.463	3.740 ^+^	1.996	−13.042 ***	2.633
Female	−16.292 ***	2.459	15.250 ***	2.298	0.058	1.599	−20.306 ***	3.449	8.074 **	2.676	−11.925 **	3.641
Male	−11.540 ***	2.511	8.554 ***	2.307	−2.528	1.650	−14.353 ***	3.473	−0.708	2.952	−14.214 ***	3.728
Household income	−0.003 ***	0.000	0.003 ***	0.000	0.000	0.000	−0.003 ***	0.000	0.001 ***	0.000	−0.001 *	0.000
Female	−0.003 ***	0.000	0.003 ***	0.000	0.000	0.000	−0.003 ***	0.001	0.001 **	0.000	−0.002 **	0.001
Male	−0.003 ***	0.000	0.003 ***	0.000	0.000	0.000	−0.002 ***	0.001	0.002 **	0.000	−0.001	0.001
Living in urban area	−9.041 ***	2.374	12.184 ***	2.191	3.596 *	1.497	−1.857	3.288	12.094 ***	2.674	9.811 **	3.529
Female	−9.233 **	3.156	9.807 **	2.968	1.335	1.982	2.724	4.471	12.060 **	3.466	14.145 **	4.706
Male	−10.261 **	3.479	15.723 ***	3.227	5.328 *	2.216	−5.973	4.787	12.286 **	4.109	6.024	5.197

* *p* < 0.05, ** *p* < 0.01, and *** *p* < 0.001; SE, standard error.
